# Study on the Effect of Micro-Force Perturbations and Temperature Fluctuation on Interferometer for the Taiji Program

**DOI:** 10.3390/s24010098

**Published:** 2023-12-24

**Authors:** Juan Wang, He-Shan Liu, Chao Yang, Ke-Qi Qi, Zi-Ren Luo, Ran Yang

**Affiliations:** 1 Center for Gravitational Wave Experiment, National Microgravity Laboratory, Institute of Mechanics, Chinese Academy of Sciences, Beijing 100190, China; wangjuan@imech.ac.cn (J.W.); liuheshan@imech.ac.cn (H.-S.L.); yangchao1@imech.ac.cn (C.Y.); qikeqi@imech.ac.cn (K.-Q.Q.); luoziren@imech.ac.cn (Z.-R.L.); 2 Taiji Laboratory for Gravitational Wave Universe (Beijing/Hangzhou), University of Chinese Academy of Sciences, Beijing 100190, China

**Keywords:** micro-force perturbation suppression, temperature fluctuation suppression, interferometry, Taiji program

## Abstract

To increase the interferometric measurement resolution in the Taiji program, we present a noise suppression method in this paper. Taking the specific micro-force perturbation and temperature fluctuation in the Taiji-1 interferometer as an example, we set up and experimentally verified the corresponding transfer function to quantify the effect of both noise sources on the interferometric results. Consistent results were obtained between the numerical and experimental results for the transfer function. It is instructive to eliminate the micro-force perturbations and temperature fluctuations during on-orbit interferometric measurement for as long as the acquisition of the force or temperature distribution of related surfaces and the corresponding transfer functions. This indicates that the method can be used for noise sensing and more in the field of noise elimination and measurement resolution improvement for future Taiji program interferometers.

## 1. Introduction

Space-borne gravitational wave (GW) detectors focus on the detection of GWs with the low-frequency region, such as Laser Interferometer Space Antenna (LISA) and the Taiji program from 0.1 mHz to 1 Hz [1,2,3,4]. The Taiji program, as the Chinese space-borne GW detection program, is required to reach a ranging resolution of 1 pm/$\sqrt{\mathrm{Hz}}$ [2,3,4]. Inter-satellite laser interferometry ranging is used to measure the distance change between two test masses and further allows for the observance of gravitational wave signals. Reaching the required precision means that key technologies need to be applied, including high-resolution laser interferometry, the gravitational reference sensor, the drag-free control system, the ultra-static satellite platform, etc. [4,5]. During in-orbit operation, the satellite can be interfered with not only by inevitable external micro-forces, such as the solar wind, solar pressure, and especially the thrust from the micro-thruster, but also temperature fluctuations, caused by the solar temperature modulations and power fluctuation of the electronic components. In particular, temperature fluctuation has been identified as the leading factor in the noise contribution at mHz frequencies, which cannot be ignored; this creates effects through two means: the movement of the test mass and the deformation of the interferometer [6].

To reduce the effect of micro-force perturbations or temperature fluctuation on the interference results, two approaches are used: one is to actively improve the structural or temperature stability, such as preliminary structural optimization for the former and a passive insulator or active control loop for the latter [7]; the other is to passively eliminate the impact of the noise sources, which can be realized by measuring the noise, establishing the transfer function of the noise sources and interference results, and subtracting its contribution from the main interferometric stream [8]. In the second method, the variable driving the noise contribution, like temperature, must be measured. Taking the LISA Pathfinder engineering model as an example, temperature sensors are distributed, and a spectral analysis method for temperature noise subtraction is proposed to establish the transfer function between temperature noise and interferometer measurements. The result shows a significant noise reduction at lower frequencies with a performance of 100 nm/$\sqrt{\mathrm{Hz}}$ at 30 μHz after temperature noise subtraction. In addition, it provides the possibility for on-ground lab experiments to compute a performance curve without the limitation of the unavoidable temperature drifts, achieving results that are close to space conditions [9]. Based on these investigations, throughout the duration of the LISA Pathfinder mission, a precision thermal diagnostic system was built and operated to analyze the effect of temperature fluctuation in different locations. This system was composed of 24 thermistors and 16 heaters to allow the precise characterization of the temperature variations and produce controlled inputs to calibrate the experimental response, respectively. In terms of performance, it achieved the required performance of 10 μK/$\sqrt{\mathrm{Hz}}$ from 1 mHz to 30 mHz [10].

However, the effect of these perturbations on the interferometer has not been considered in the Taiji program to the best of the authors’ knowledge. To further improve the interference ranging resolution, an analysis method is proposed in this paper to evaluate and eliminate the effect of both micro-force perturbations and temperature fluctuation. It consists of discovering the relationship between the noise sources and the interference result through the simulation process, followed by subtracting its influence from the final optical path result. This method has been introduced to estimate the effect of thrust on the optical path length difference fluctuations of the interferometer for the Taiji-1 whole satellite model, which is the technology demonstration satellite as the first step for the Taiji program [11]. The Taiji-1 satellite was launched in 2019 and demonstrated to be capable of 100 pm/$\sqrt{\mathrm{Hz}}$ covering the measurement band from 10 mHz to 1 Hz for interferometer measurement sensitivity, i.e., interferometer stability, and ±5 mK/$\sqrt{\mathrm{Hz}}$ for temperature stability [12,13]. It should be noted that the method to estimate the effect of thrust on the interference results lacks experimental verification, which needs to be considered further.

To experimentally verify the feasibility of the noise suppression method, a backup optical bench of the Taiji-1 interferometer is employed in this paper. The analysis of all possible micro-force perturbations and temperature fluctuation is complex because it is hard to simulate the on-orbit environment. Fortunately, for the Taiji-1 interferometer, the number of contact surfaces between the interference platform and satellite is well-defined, which allows for separate building of the transfer function for each contact surface provided a given noise source. As a simplified analysis, we pay attention to the specific perturbation situation where the micro-force perturbation noise or temperature fluctuations are subjected to only one of the contact surfaces in the Taiji-1 interferometer model.

This paper is organized as follows. We describe the establishment of the transfer function between the force and interferometer noise and the experimental verification results in Section 2. The same method used for temperature fluctuation is presented in Section 3 together with the noise suppression effect, followed by the conclusions in Section 4.

## 2. Micro-Force Perturbation Noise Analysis

### 2.1. Establishment of Transfer Function between Force and Interferometer Noise

**Figure 1:**
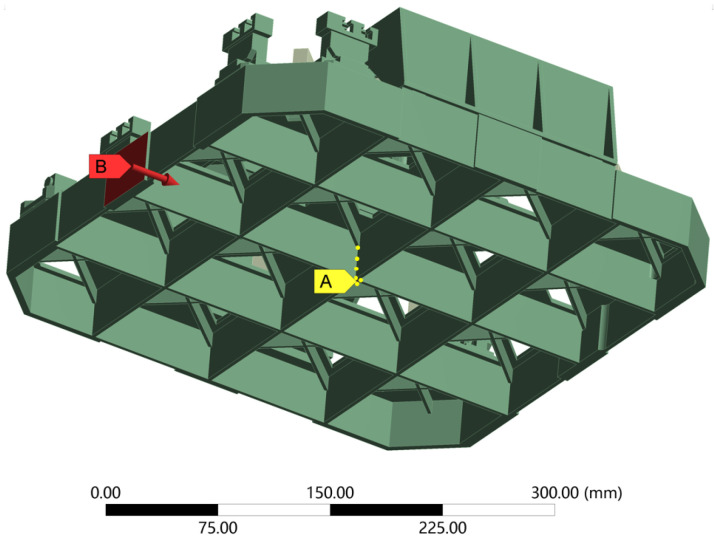
A sketch of force and constraint arrangement.

**Figure 2:**
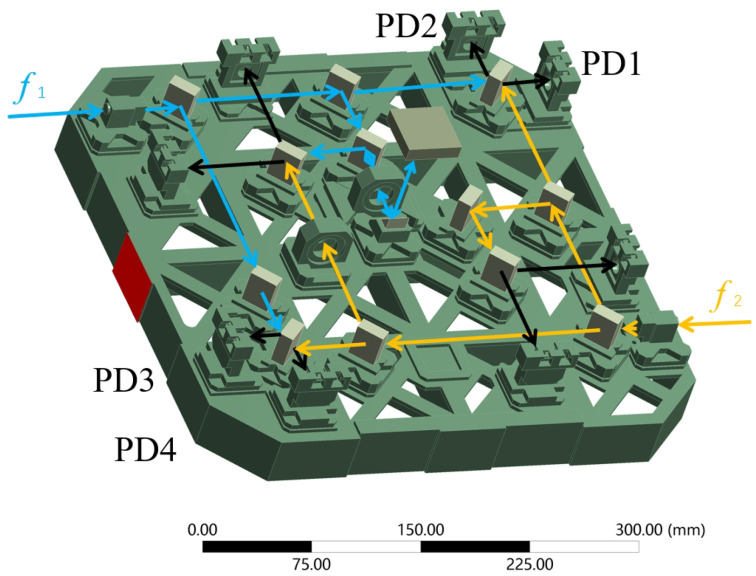
A schematic view of the optical paths in the Taiji-1 interferometer.

The objective of this section is to experimentally quantify the interferometer result fluctuation generated by the force perturbations. It is difficult to recreate the true in-orbit loading conditions when conducting a ground experiment. Hence, we simplified the model, the loading force, and the loading constraints to set up the transfer function between the force and the optical path signals. The detailed introduction of the Taiji-1 interferometer has been presented in the literature [11], and a brief simulation-related introduction of the interferometer model and optical path layout are given in Figure 1 and Figure 2, respectively.

As indicated in Figure 1, the simplified Taiji-1 interferometer model is constrained by the 16 fixed nodes (A) located on the bottom center of the interferometer platform to lessen the effect of the friction, which is far greater than the given force with a magnitude of mN or even μN between the interferometer platform and the optical substrate. In addition, the specific part (B, marked in red) is chosen to be subjected to the given force, because it is one of the six surfaces rigidly connected to the whole satellite. Here, the force is dependent on time and is assumed to be:

(1) $$F=\frac{A}{2}+\frac{A}{2}\cdot sin\left(2\pi \cdot f\cdot t\right),$$

where *A* is the peak-to-peak value with the unit of mN, and *f* is the frequency. The influence of force disturbance on the final interference result is not related with frequency, since the noise transmission mechanism for force disturbance is defined by the system structure. As a consequence, *f* = 0.5 Hz is taken as an example in this paper to verify the effectiveness of the simulation modeling method.

A schematic view of the optical paths in the Taiji-1 interferometer is shown in Figure 2, where the lens bases, detector supports, optical fiber collimator, one prism, and twelve beam splitters are mounted on the platform directly. As a result, deformation occurs in the key optical components (one prism and twelve beam splitters), which are cemented to the frames. The node coordinate of each component can be derived through structural simulation.

The deformations in the optical components will influence eight interference signals (the black light ray), separately detected by the eight photodetectors. The interference signal with the difference frequency $f_{1}$–$f_{2}$ in photodetectors PD1, 2, 3, and 4 reflects the optical path difference between two optical rays and further the stability fluctuation of the interferometer. It provides a way to quantitatively analyze the effect of the micro-force perturbation on the interferometer stability, which can be confirmed by the subtraction result of the signal between photodetector 1 or 2 as well as photodetector 3 or 4 as a function of time. In general, the optical simulation can be realized by establishing a system model, creating a light source, ray tracing, and system performance analysis. This simulation represents performing the surface fitting of the optical components; creating a laser with a wavelength of 1064 nm; simulating the optical path track and the combination of two beams of light; and exporting the optical path difference data of the four interference optical paths at each time step.

**Figure 3:**
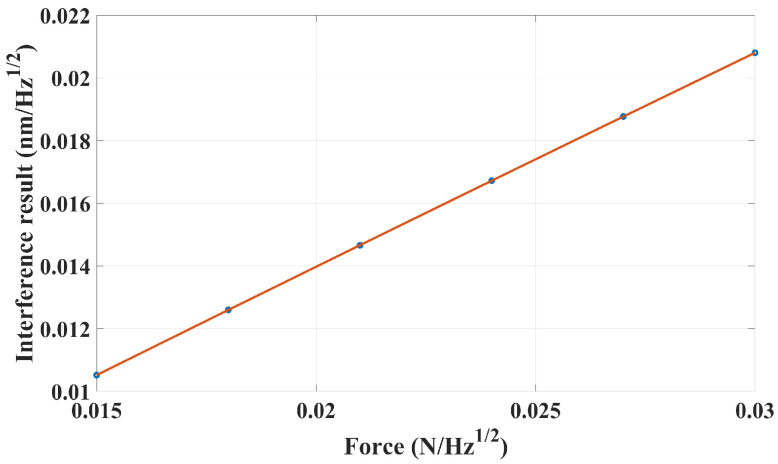
A scatter diagram and fitting curve of the simulation results.

Six different force conditions are simulated, where *A* is assigned with values of 0.5, 0.6, 0.7, 0.8, 0.9, and 1.0. In each case, the directional deformation of all nodes on the thirteen optical components can be exported and the new node coordinates can be calculated to form a new surface of the components every 0.2 s, i.e., a sampling frequency of 5 Hz. The eight interference signals can be obtained every 0.2 s after the light tracing process with a series of parameter settings, such as the light wavelength, reflective index (1.45) of the components, as well as the coating properties. The corresponding subtraction result between photodetectors 1 and 3 is extracted to display the influence of the micro-force perturbation. To be consistent with the following experimental validation, the sampling numbers in the simulation are 36,000, which corresponds to a duration of 7200 s. Finally, the amplitude spectrum density (ASD) value of the reference results (PD1–PD3) in each case can be obtained at a frequency of 0.5 Hz, corresponding to the different peak-to-peak values of the micro-force perturbation. In this paper, an ASD value of measured signal is selected to the transfer function analysis because it is related with time and helps magnify the weak signal. A scatter diagram and fitting curve are given in Figure 3.

The expression of the fitting curve is represented in Equation (2),

(2) y=0.02056·F+0.0002562,

where *y* is the interference result (nm/$\sqrt{\mathrm{Hz}}$) and *F* denotes the peak-to-peak value of the micro-force perturbation (mN). Moreover, the quality of the fitting curve has a $R^{2}$ value of 1, which demonstrates a good linearity. The coefficient of determination, the $R^{2}$ value, is used in this paper to reflect the goodness of fit.

### 2.2. Experimental Verification of the Transfer Function

**Figure 4:**
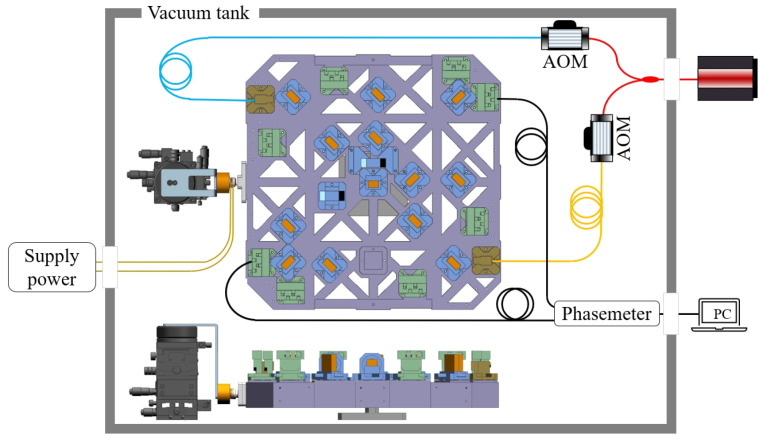
Schematic diagram of the experiment system (above: top view; bottom: front view).

Consistent with force application and the constraint conditions settings mentioned above, we experimentally verified the results of Equation (2), and the set up is shown in Figure 4.

The system consists of two parts: one is located inside the vacuum tank, and the other is located in the air to be controlled manually. As for the module inside the vacuum tank, a top view is given to show the whole system structure, including the interference optical path, the force application equipment, and the signal postprocessing process. A laser with a wavelength of 1064 nm is provided by the stable laser systems (SLS-1064-1000-1, with frequency stability better than 550 Hz/3 h) in a mW magnitude for the actual optical power after the attenuation. After the acousto-optic modulator (AOM), the two beams of the two lasers with different shifted frequencies enter the interference optical system. The force application equipment, located on the left side of the interferometer platform, is the assembly of a multiturn coil and a permanent magnet. It can provide the force with the needed peak-to-peak value and frequency using a USB-6289 by National Instrument, as explained in Section 2.2.1. Additionally, to measure the influence of the micro-force perturbation, the interference results from photodetectors 1 and 3 are received and post-processed in the phasemeter, where a digital-phase lock-loop technique is performed with the phase measurement resolution of 0.5 μrad/$\sqrt{\mathrm{Hz}}$ [14,15]. As a supplement, the front view located at the bottom of Figure 4 indicates that the center of the optical platform is lifted by the support to reduce the friction between the interferometer platform and the optical substrate.

#### 2.2.1. Calibration of the Force Application Equipment

The application of the micro-force application equipment is the key issue, and it can be solved by the introduction of the electromagnetic force created by the assembly of a multiturn coil and a permanent magnet. As shown in Figure 4, the permanent magnet is fixed on the frame and controlled with a six-degrees-of-freedom displacement table. On the contrary, the multiturn coil, sleeved on a support made of engineering plastic, is stuck to the assigned surface of the platform. It has been proposed that the created electromagnetic force is proportional to the current in the multiturn coil [16], and the direction of the created force is along the axis of the coil. This is essential for calibration of the force application equipment.

**Figure 5:**
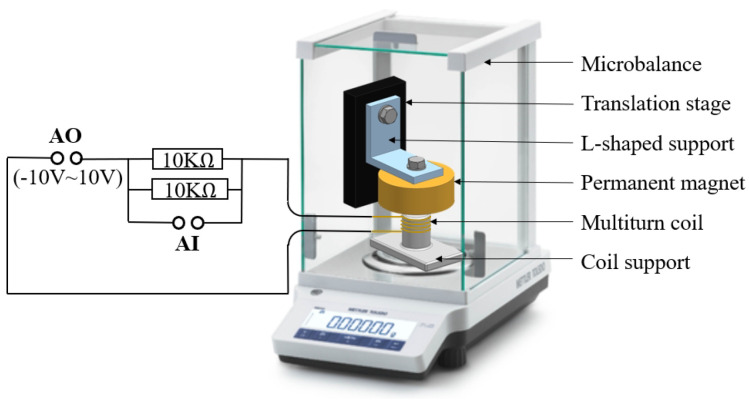
The diagram sketch of the microbalance method.

The microbalance method is used in this paper to calibrate the relationship between the electromagnetic force and the voltage of the coil. The diagram sketch of the microbalance method is illustrated in Figure 5 [16]. The circuit diagram on the left is the circuit connection mode of the USB-6289, where the analog output (AO) is used for the voltage value setting and the analog input (AI) is for the voltage value reading. The force application equipment is in a microbalance ME55 by METTLER with a resolution of 10 μg and a typical repeatability of 20 μg. The multiturn coil is located on the base of the microbalance with an unadjusted position. To control the relative position between both, the permanent magnet can realize movement in three translational degrees of freedom through the translation stage, which is fixed inside the microbalance. Moreover, the coil is controlled by the USB-6289 outside the microbalance, and then the electromagnetic force between the coil and the permanent magnet is formed because of the magnetic field generated from the permanent magnet according to the Faraday law of electromagnetic induction. The electromagnetic force can be read from the change in the microbalance mass reading (mass × 9.8 N/kg).

Specifically, the relative position between the coil and permanent magnet can be adjusted to reach a maximum mass reading (the electromagnetic force). We recorded the maximum mass readout for the analog output voltages of 0, 0.1, 0.2, 0.4, 1, 2, 4, 8, and 10 V. Finally, the calibrated relationship between the average force and the voltage of the force application equipment after three groups of repeated experiments is

(3) $$F_{c}=0.1237624\cdot U_{c}+3.802\times 10^{-4},$$

where $U_{c}$ denotes the analog output voltage constant in V, and $F_{c}$ is the produced average electromagnetic force in mN. Meanwhile, the curve fitting R^2^ value of Equation (3) is 1. For the force application equipment, the maximum force is about 1.238 mN when the analog output voltage constant is 10 V.

#### 2.2.2. Obtained Transfer Function Based on the Experiment

Since the linear coefficient between the voltage and the related force has been obtained, we can apply the varied force with a sinusoidal wave to the assigned surface, as shown in Figure 4. The voltage frequency of the force application equipment is set to 0.5 Hz and the peak-to-peak value is 5 V (6, 7, 8, 9, or 10 V) with the same expression form in Equation (1). To be consistent with the simulation time, 432,000 interference data points are exported from photodetector 1 and 3 each, because the sampling frequency of the phasemeter is 60 Hz. Then, the spectral response curve of the interference result (subtraction result between photodetectors 1 and 3) can be obtained by the fast Fourier transform when the voltage peak-to-peak value is 5 V (6, 7, 8, 9, or 10 V), and the corresponding interference result at 0.5 Hz of each voltage peak-to-peak value is used to establish the transfer function between the voltage and the interference result.

**Figure 6:**
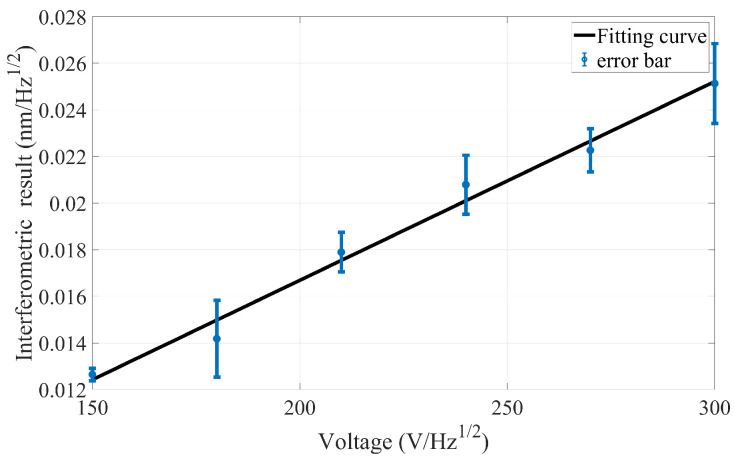
The fitting curve of the interference result and voltage.

To enhance the accuracy of the experimental results, four groups of repeated experiments were carried out for the above different peak-to-peak values. As a result, the average interference result at 0.5 Hz of each voltage peak-to-peak value is given in Figure 6, where the corresponding error bar and the fitting curve are also provided.

This indicates that the experimental fitting curve is within the range of the error bar, which means that the experimental fitting curve obtained is effective in the repetitive experiment. Furthermore, the fitting curve between the average interference results and the analogous output voltage peak-to-peak value is:

(4) y=0.002559·U−0.0003754,

where *U* is the peak-to-peak value of the sinusoidal voltage. Meanwhile, *y* is the maximum spectral response of the interference result at a frequency of 0.5 Hz. The quality of the fitting curve has a R^2^ value of 0.9874.

Substituting Equation (3) into Equation (4), we will obtain the final transfer function considering the micro-force perturbation and the interferometer stability:

(5) y=0.02067·F−0.00303,

where *F* is the peak-to-peak value of the electromagnetic force in mN.

### 2.3. Discussion

Comparing the transfer functions obtained from the simulated and experimental processes, Equations (2) and (5), it should be noted that the constant term should be 0, i.e., no force perturbation and no interference error, and the error is related with the settings of the simulation and the experimental setup. However, we have quantitatively demonstrated the agreement between both first-order coefficients with an error of 0.53%. The effectiveness of the simulation modeling method mentioned in this paper is proven through four groups of repeated experiments. These results show that a follow-up study on the effect of the force distribution belonging to all contact surfaces on the interferometer is feasible in the future of the Taiji program.

In addition, several reasons that follow provide explanations for the error in the fitting linear coefficient.

At first, the simulation process is simplified compared to the experimental process. Except for the simplified mechanical model and the restrictions, it does not take into account the change in the deformation of the photodetector position in the optical light tracing procedure. Moreover, the influence of the components’ refractive index fluctuation caused by the nonuniform stress distributions is not contained in our simulation analysis because of the complicated stress distribution.

Secondly, the error can be attributed to the resolution of the force application equipment. The relationship obtained in the calibration procedure is limited by the accuracy of the microbalance reading and the external environment interference in the microbalance method. Additionally, the influence of the slight position deviation between the assembly of a multiturn coil and a permanent magnet on the electromagnetic force exits in the validation experiments. We ignored the effect of this part because it is almost the same for the magnitude of the electromagnetic force when there is a short distance between both components under the same current [17].

Thirdly, the effect of the ambient temperature fluctuation during the experiment cannot be ignored. Except for the reading error from the temperature drift in the calibration process, thermal stress occurs in the interferometer platform and final optical components due to the thermal conduction and radiation from the ambient and electrical components temperature fluctuation, and it will result in the extra interference noises.

## 3. Temperature Fluctuation Noise Analysis

### 3.1. Preparation of Experimental System

#### 3.1.1. Design of Experimental System

**Figure 7:**
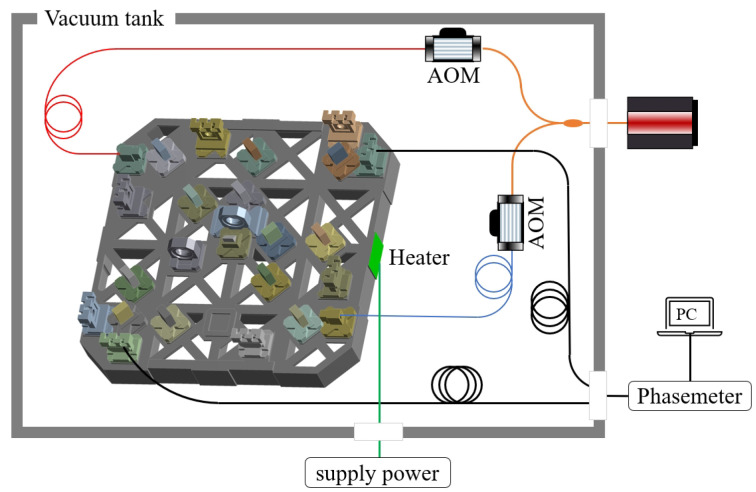
Schematic of experimental system for thermal induced effects analysis.

For temperature fluctuation analysis, to establish the relationship between thermal drift noise and interferometric measurement results, the distribution of thermal noise must be considered first. Based on the connection between the Taiji-1 interferometer and other satellite components, the six surfaces on both sides of the interferometer will transmit thermal disturbances to the interferometer’s optical platform. Therefore, one of the surfaces is taken as an example to analyze the impact of thermal drift noise on interferometric measurement. The overall system diagram is shown in Figure 7.

The temperature fluctuation is provided by a heater, which is pasted on the surface of the interferometer platform and controlled by the power on/off time of the external power supply for different amplitudes and periods. The impact of noise on interferometric measurement is read out through the optical system shown in the figure, similar to the force disturbance experimental verification system. The laser, identical to the one used in Section 2, is divided into two beams through AOMs. Two laser beams with a 1.6 MHz differential frequency enter the interferometer platform through two collimators each and finally encounter in the photodetector, which converts the interference signal into an analog signal and sends it to the phasemeter (with a phase measurement accuracy better than 0.5 μrad/$\sqrt{\mathrm{Hz}}$). In the whole system, the components inside and outside the vacuum tank are connected through the flange.

#### 3.1.2. Establishment of Experimental System

**Figure 8:**
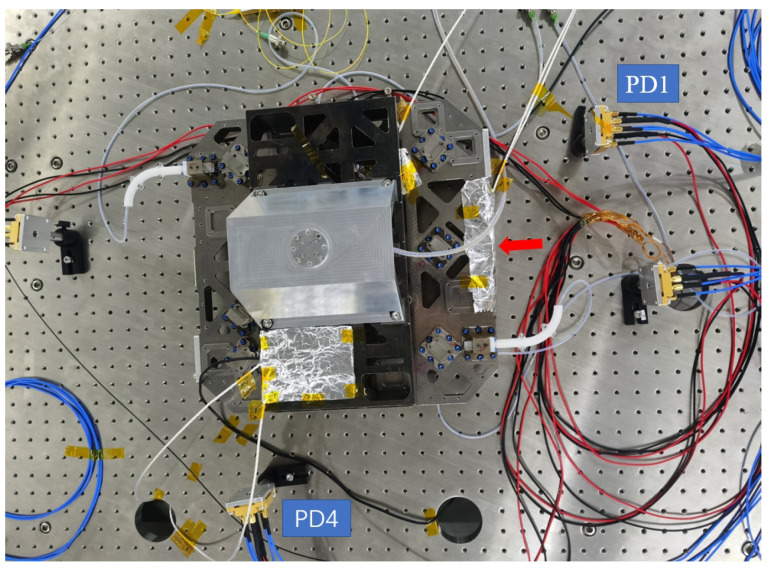
Photograph of the experimental equipment.

The experimental setup follows the schematic layout of Figure 7 and is shown in Figure 8.

To eliminate the influence of thermal convection and simulate the spatial environment, the experiment was conducted in a vacuum environment. The heat source is located on one of the surfaces on the right side of the interferometer (marked as an arrow), and the temperature sensor is distributed in different locations of the interferometer for more accurate measurement of temperature distribution, all covered with aluminum foil to reduce the direct radiation effect of the heat source. Therefore, analyzing the impact of temperature fluctuation noise on interference measurement during the heat conduction process is the focus. The bottom of the interferometer is suspended through a support frame to reduce the heat conduction between the interferometer and the substrate. To evaluate the stability of the interferometer under the influence of thermal noise, the interference results of two equal arm length optical paths were taken in this analysis, namely the difference between interference results (PD1–PD4). Therefore, the response of the interferometer platform mainly comes from the temperature fluctuations of the heat source, which means that the interferometric measurement results can reflect the thermal drift noise.

#### 3.1.3. Design of Temperature Fluctuation Load

**Figure 9:**
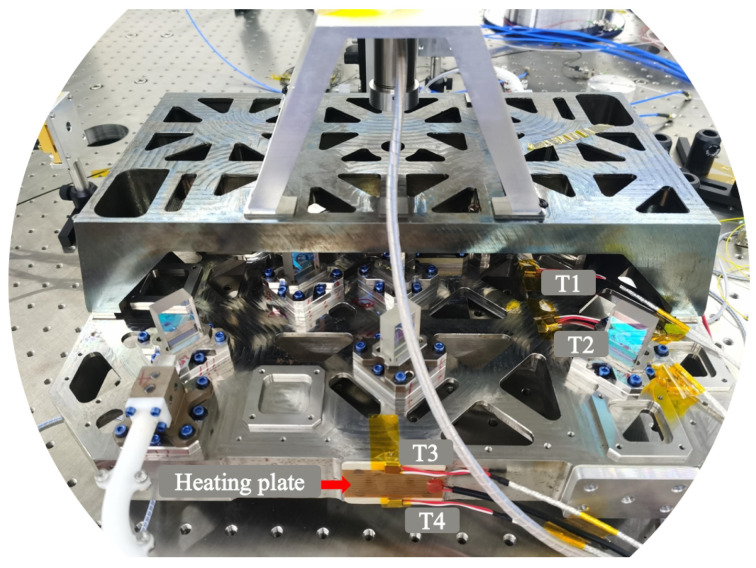
Photograph of optical bench with heat source and thermometer.

Compared to the loading and unloading of force, the loading and unloading of thermal loads are related to material properties, structural design, and environment, making it difficult to achieve thermal disturbance loading with arbitrary sinusoidal fluctuations. Therefore, it is necessary to design the thermal disturbance load of the heater to ensure that the same thermal load is used in both simulation and experiment. The distribution of the heater and the four temperature sensors is shown in Figure 9.

The application of the temperature load is achieved through a Kapton heating plate with a nominal voltage of 12 V and a nominal power of 7 W. The application of the temperature load can be controlled by adjusting the input voltage of RIGOL’s DP800 DC power supply. For example, the input high level (12 V) is used for heating, and the input low level (0 V) is used for cooling (not heating continuously). The power supply duration and number of cycles of the input voltage represent the period and working time of the temperature fluctuation, respectively. Temperature sensors T1 and T2 are located on the interferometer platform, while T3 and T4 are located near the heater. PRTs (Platinum resistance thermometers) are used as temperature sensors and they have a sensing accuracy of 0.1 °C between −20 °C and 60 °C, with a resolution of 0.05 °C. The data output rate is 0.5 Hz (the amount of data per unit time, *Fs = N/T*, where *N* represents the total amount of data and *T* represents the duration). The period of the heat source temperature fluctuation is 22 min.

**Figure 10:**
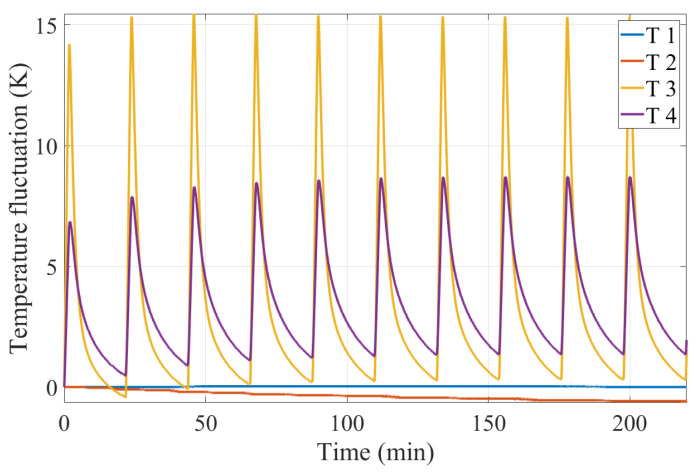
Relative temperature change in response to the heat source over a period of 22 min.

Since the interference measurement resolution is related to the thermal stress stability of the interferometer, it is necessary to maintain the stability of temperature fluctuations as much as possible, that is, to cool down to the room temperature after heating. Therefore, considering the consistency between the experimental verification process and simulation process, the temperature fluctuation is loaded with a period of 22 min (1320 s), including 2 min for heating (with a heat flow power of 7 W) and 20 min for cooling (with a heat flow power of 0), 10 cycles in total. The data output rate is 0.5 Hz and the duration is 13,200 s. A total of 6600 temperature points were sampled and the measured relative temperature fluctuation of sensors after subtracting respective initial temperature is shown in Figure 10. The temperature fluctuations of T3 and T4 show an expected thermal load, while T1 and T2 show a different trend. It can be attributed to the poor contact between sensor and interferometer and the limited accuracy of sensors.

### 3.2. Establishment of Transfer Function between Temperature Fluctuation and Interferometer Noise

#### 3.2.1. Analysis of Temperature Field

**Figure 11:**
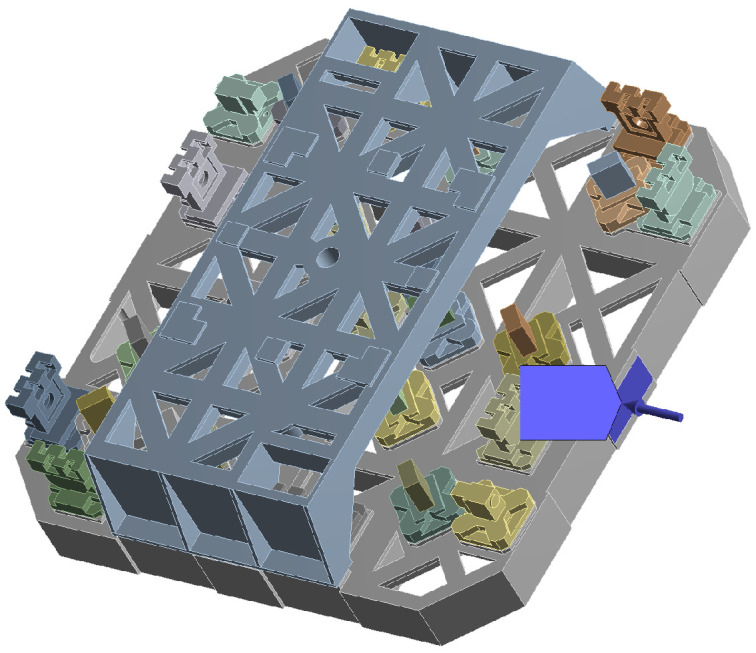
Schematic of the surface of thermal disturbance.

Similar to the micro-force perturbations analysis, temperature loads are applied to the specific contact surface between the interferometer and the satellite. The size of the contact surface is 30 mm × 50 mm, while the size of the heating plate used in the experiment is 25 mm × 50 mm. Therefore, in the transient thermal analysis, a print surface with a size of 25 mm × 50 mm was utilized as the target surface, as shown in Figure 11. Similarly, during the simulation process, heat flows of 7 W and 0 W were applied to the print surface for 2 and 20 min, respectively, each being one period.

**Figure 12:**
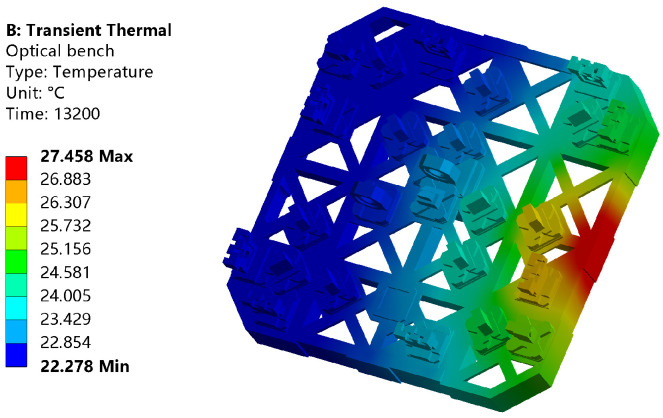
Temperature distribution of optical bench at 13,200 s.

The initial temperature of the analysis process is set at 22 °C. It consists of 660 analysis substeps, which means that a temperature field distribution can be obtained every 20 s and the corresponding sampling frequency is 0.05 Hz. As an example, the temperature field distribution of the entire optical platform at 13,200 s is shown in Figure 12.

The color bar on the left displays the maximum and minimum temperature values on the platform at this time. The result shows that the temperature distribution of the optical bench is different. The further away from the heat source, the lower the temperature value. There are also temperature gradients far away from heat sources, which are not shown in the cloud map limited by the display accuracy. In addition, due to the asymmetry of the entire platform structure, the temperature distribution of the entire platform is not completely symmetrical.

#### 3.2.2. Analysis of Thermal Deformation

**Figure 13:**
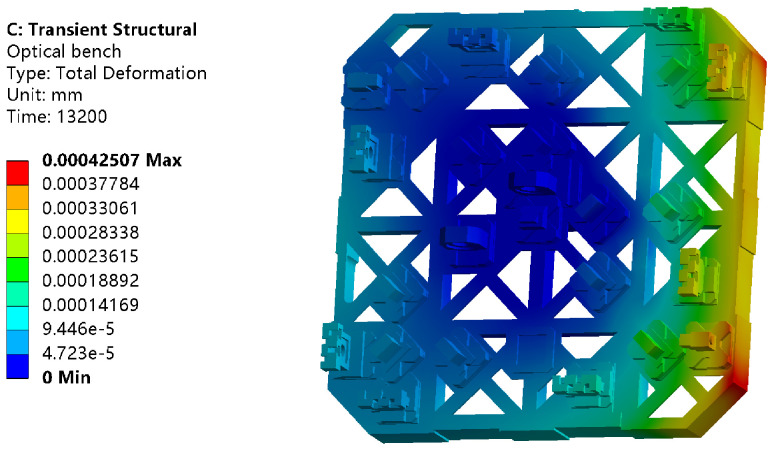
Total deformation of optical bench at 13,200 s.

From the above temperature field simulation, it indicates that the temperature distribution of optical components changes over time due to the influence of heat sources. Additionally, there is a fixed constraint of a support frame at the bottom of the optical platform. The optical bench is made of invar steel and the optical components are made of fused silica, i.e., with a different thermal expansion coefficient. Therefore, changes in the temperature field inside the elastic continuous medium can cause thermal stress, which in turn leads to a deformation. Transient structural analysis was used to analyze the deformation results of optical components under different temperature fields. During the temperature field analysis process, the analysis duration is 13,200 s and the analysis number of substeps is 660, with the same sampling frequency of 0.05 Hz. The interferometer is subjected to temperature load only and is fix constrained at 16 points at the center of the bottom. The simulated results show a varied deformation with the change in temperature field. As an example, the total deformation of the entire optical bench at 13,200 s is shown in Figure 13.

Due to the presence of fixed constraints, the deformation of the middle part of the optical bench is 0, and the deformation near the heat source is greater than that found far away from the heat source. The areas with the greatest deformation are concentrated in the upper right and lower right of the optical bench, as the radiation process was not considered in this simulation process. Finally, the deformation of the 13 optical components within the platform varies and exhibits periodic changes over time. In response to the deformation requirements for 13 optical components involved in the subsequent optical simulation process (including 24 optical surfaces, totaling 1857 nodes), the coordinate values of the X, Y, and Z components corresponding to each node in each substep are derived.

#### 3.2.3. Establishment of Transfer Function

The interference measurement noise caused by temperature disturbance is positively correlated with the initial optical path length, refractive index, and thermal expansion coefficient. For the same temperature fluctuations, the optical path noise caused by deformation of the invar substrate is about one order of magnitude larger than that of the optical components. In addition, considering that the heat source is directly applied to the side of the substrate in this simulation, the temperature fluctuation amplitude of the substrate is larger than that of the optical components, which will lead to the dominant optical path noise of the invar substrate. Therefore, in this optical simulation, the optical path noise caused by the refractive index of the components is ignored, and the main focus is on the optical path changes caused by the thermal deformation of the invar substrate and the optical components. Similar to the optical simulation process in micro-force perturbation noise, it involves exporting the node coordinates of the optical components, reconstructing the optical surface, ray tracing, and extracting the real-time optical path results from two photodetectors of the interferometer.

**Figure 14:**
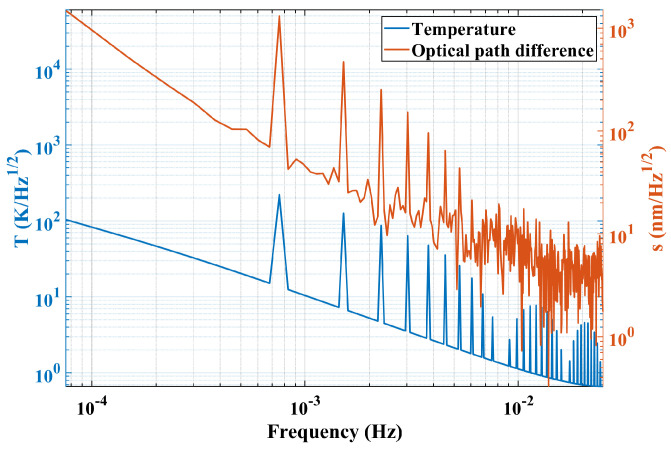
ASD curve of temperature and optical path difference in the simulation process.

The data postprocessing of the obtained interference result is as follows: through optical simulation, the interference optical path results of detector 1 and detector 4 can be obtained separately, as described in Section 2.1; both can be subtracted to obtain a matrix of 660 × 1 to reflect the stability of the optical platform; the ASD of the subtracted results (y) can be obtained [18]:

(6) $$ASD=\sqrt{\frac{{2|fft\left(y\right)|}^{2}}{F_{s}N}},$$

where *y* represents the time domain result after subtraction, such as the optical path difference result or temperature value, $F_{s}$ = 0.05 Hz is the sampling frequency, *N* = 660 is the number of sampling points, and fft is the Fourier transform. The ASD curve of temperature and optical path noise is shown in Figure 14.

The corresponding peak value in the special frequency of both curves can be obtained in Figure 14, like 0.0007576 Hz and frequency multiplication. The response frequency of the interference result is the same as that of the temperature fluctuation with a value of:

(7) $$f=\frac{1}{22*60}=0.0007576\;\mathrm{Hz},$$

since the period of the temperature fluctuation is 22 min. Moreover, the high-frequency response of optical path difference curve is submerged in the noise, which is caused by the limited data number and simulation resolution.

**Figure 15:**
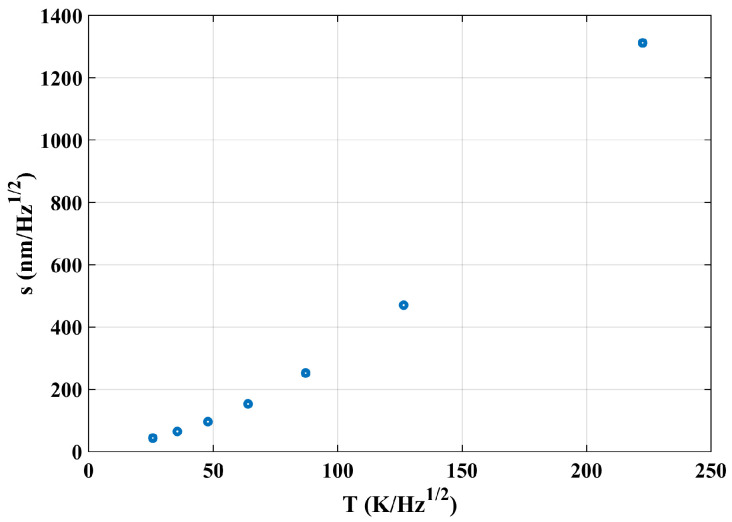
Scatter diagram between the optical path noise peak value and the temperature fluctuation peak value.

Taking the peak value of both temperature fluctuation and response optical path noise at the same frequencies in Figure 14, a scatter plot of interference optical path noise (s) with temperature fluctuations (T) was obtained and shown in Figure 15.

Theoretically, the influence mechanism of temperature on interference measurement is independent of the frequency and only decided by the system’s thermal-related coefficients, like the thermal expansion coefficient, which means the coupling relationship should be linear. However, the optical path difference noise in Figure 15 shows a non-linearly increasing trend with the increase in temperature, and it can be explained as follows.

Taking the heat conduction equation for a uniform semi-infinite object as an example, its one-dimensional heat conduction differential governing equation is [19]

(8) $$\frac{\partial \theta (x,\tau )}{\partial \tau }=a*\frac{\partial^{2}\theta \left(x,\tau \right)}{\partial x^{2}},$$

where θ(x,τ) is the temperature at position *x* and moment τ, and *a* is the thermal diffusivity.

When the boundary condition is time-dependent and simplified to

(9) $$\theta \left(0,\tau \right)=A_{w}*cos\left(2\pi f\tau \right)$$

on x = 0, and

(10) θ(∞,τ)=0

on x→∞. The derived temperature field is

(11) $$\theta \left(x,\tau \right)=A_{w}*e^{-\sqrt{\frac{\pi f}{a}}x}cos\left(2\pi f\tau -\sqrt{\frac{\pi f}{a}}x\right).$$

Simplifying the analysis process and assuming only the freedom expansion under heat conduction, the thermal deformation is

(12) $$s\left(x,\tau \right)=\alpha *\theta \left(x,\tau \right)=\alpha *A_{w}*e^{-\sqrt{\frac{\pi f}{a}}x}cos\left(2\pi f\tau -\sqrt{\frac{\pi f}{a}}x\right),$$

where α is the thermal expansion coefficient.

In principle, the relationship between the interference result s(x,τ) and temperature fluctuation θ(x,τ) is linear. The nonlinear coupling in Figure 15 is due to the choice of parameters: the peak value of the temperature $A_{w}$ and the interference result $\alpha *A_{w}*e^{-\sqrt{\frac{\pi f}{a}}x}$. As a result, the coupling coefficient at any position is $\alpha *e^{-\sqrt{f}}$ after normalization processing and it shows a nonlinear relationship.

The final transfer function can be obtained through fitting a quadratic curve as follows:

(13) $$s=0.02281*T^{2}+0.7851*T+7.844$$

The $R^{2}$ value is 1. When the peak response of temperature is 0, the corresponding peak response of optical path noise should be 0. The constant term is determined by the initial and boundary condition values and does not affect the mechanistic understanding of the temperature fluctuation. Therefore, only the first and second terms are considered in the following experimental verification, ignoring the constant terms of curve fitting.

### 3.3. Experimental Verification

According to the experimental setup in Section 3.1, the interference phase signals of detectors 1 and 4 are collected to analyze the response of interferometric signals to the temperature noise. Due to the working time of the thermal load being 13,200 s and the sampling frequency of the phasemeter being 20 Hz, the result of the optical path difference after the subtraction of the interference signals (PD1–PD4) is a 264,000 × 1 matrix.

**Figure 16:**
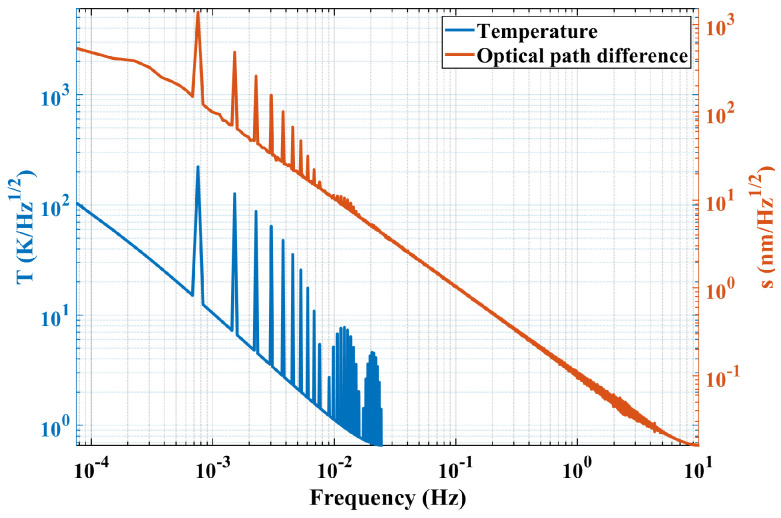
Frequency domain displacement noise result in the experimental process (temperature ASD curve is from the simulation).

Similar to the simulation process, the amplitude spectral density curve from the experimental result is used for frequency-domain post-processing analysis. Both amplitude spectral density curves are given in Figure 16, where all coordinate axes are given in logarithmic form and the temperature ASD curve is the same as the one in Figure 14.

The measurement band of temperature is relatively narrow for the reason that the temperature output rate is 0.05 Hz and the interference signal output rate is 20 Hz, while the measurement band is

(14) $$f=0:\frac{1}{T}:\frac{N-1}{T},$$

where f represents the frequency, T is the analysis duration, and N is the number of data points.

**Figure 17:**
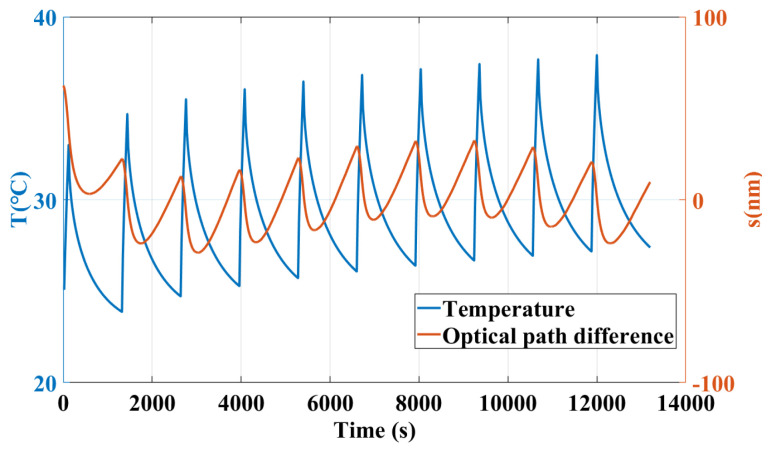
Time domain displacement noise results in the experimental process.

As a supplement, a segment of time series measurement results is shown in Figure 17.

**Figure 18:**
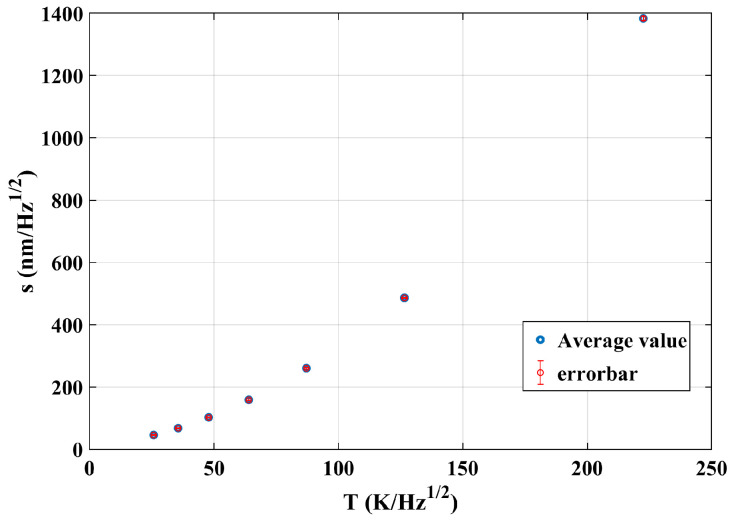
Scatter diagram between the optical path noise peak value and the temperature fluctuation peak value from the measurement data.

In order to establish the transfer function relationship between the temperature fluctuation noise and the optical path noise, three sets of experiments were conducted. Taking the results in Figure 16 as an example, the peak values of both curves corresponding to the seven intrinsic frequencies are obtained. Finally, the average values (s) of three sets of experiments at each intrinsic frequency can be obtained, and the relationship is shown in Figure 18.

The optical path noise exhibits a nonlinear trend with temperature disturbances, and the error of repeated experiments is very small. Through polynomial fitting, the final relationship between optical path noise and temperature can be obtained as follows:

(15) $$s=0.02488*T^{2}+0.6071*T+15.7$$

The $R^{2}$ value of the curve is 1. The nonlinear relationship between temperature fluctuation and optical path noise has been verified, and the feasibility of establishing a transfer function is verified through thermal disturbance simulation, ray tracing, and spectral analysis. However, a disagreement between the transfer function relationship obtained from simulation and experiment still exists, which is attributed to model and thermal environment simplification during the simulation modeling process.

### 3.4. Discussion

**Figure 19:**
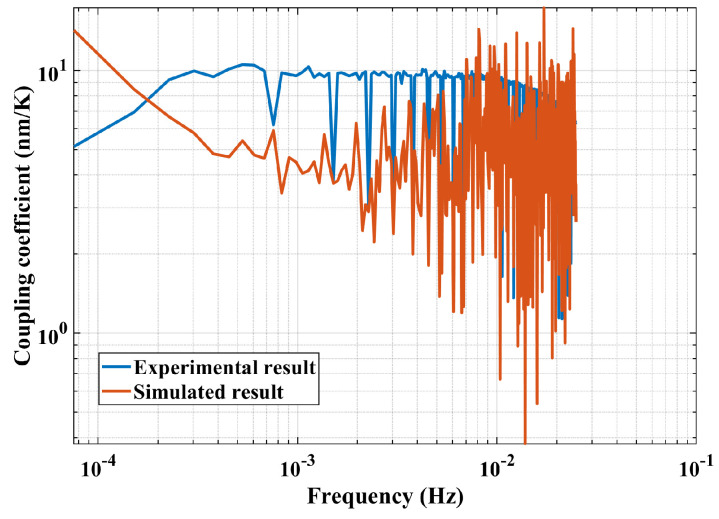
Comparison of coupling coefficients from experimental and simulated result.

Except for the verification of transfer function establishment method, the coupling coefficient of the interference result and temperature fluctuation through the whole measurement band is measured with respect to the same temperature and shown in Figure 19.

The coupling coefficient curves indicate that it is at the scale of 1 nm/K throughout the measured frequency range. Additionally, the dips/peaks from both curves being not at the same frequencies implies that there are errors in the estimated coefficient from the simulation except for the intrinsic frequency. This indicates that the effectiveness of simulation modeling is related with the accurate judgment of the noise source.

**Figure 20:**
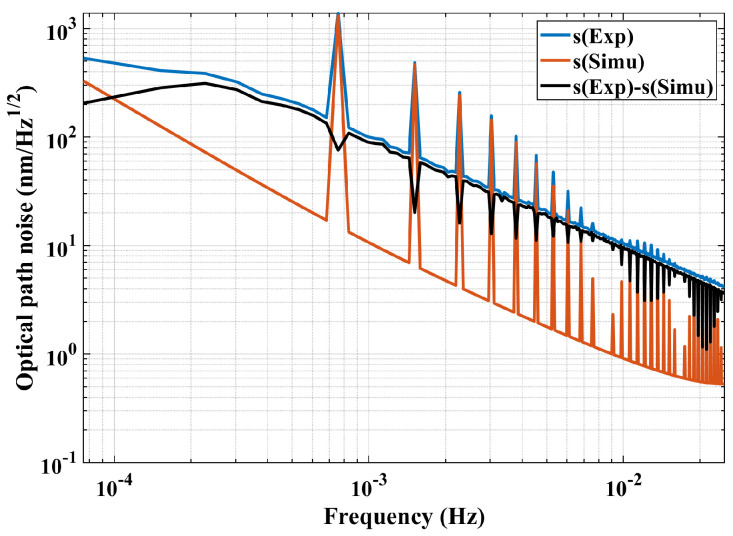
The suppression result of the thermal-induced effect.

To verify the effectiveness of the transfer function in eliminating thermal noise, thermal noise can be brought into the transfer function, ignoring constant terms, to obtain the interference result s (Simu). Then, it is subtracted from the optical path noise s (Exp) measured in the experiment to obtain the optical path noise s (Exp)–s (Simu) to complete the thermal noise elimination. The optical path noise spectrum curve obtained from the first set of experimental results is shown in Figure 20.

Due to the limitation of temperature sampling points, only the low-frequency interference signal response is displayed in the figure. It is suggested that the transfer function obtained through simulation can effectively reduce the optical path noise in the experimental results, especially at the frequency points where thermal noise acts. However, the reduction effect of optical path noise varies at different frequency points. The analysis is as follows: Firstly, the thermal stress distribution of the platform itself is complex when it is locally heated to high temperature, since the internal stress release of interferometer platform is completed at room temperature. Secondly, only the influence of heat conduction is considered in the simulation process without considering the influence of thermal radiation. Thirdly, some points are not considered in the simulation process, such as the different thermal expansion coefficients of objects such as optical components, glue and substrate inside the interferometer.

At present, the interference measurement accuracy of the noise suppression curve is about 100 nm/$\sqrt{\mathrm{Hz}}$ from 0.1 mHz to 20 mHz, which seems to be far from the accuracy requirements of the pm/$\sqrt{\mathrm{Hz}}$ level proposed by the final Taiji plan. It can be explained as follows: (1) Higher environmental thermal stability (about $10^{-5}$ K/$\sqrt{\mathrm{Hz}}$) and data processing accuracy is necessary for the interference measurement accuracy of the pm/$\sqrt{\mathrm{Hz}}$ level. Currently, a temperature insulation system has not been established during the experimental process, except for the vacuum tank. Moreover, additional heat sources were introduced to establish the system transfer function, resulting in a relatively high background thermal noise in the system. (2) The material performance and installation accuracy of the Taiji-1 interferometer are designed to be 100 pm/$\sqrt{\mathrm{Hz}}$ in the frequency range of 10 mHz–1 Hz [12]. Hence, it is necessary to improve the interferometer design (higher structure stability or lower thermal expansion coefficient), installation accuracy, environmental stability, and data post-processing level to reach the requirement of the final measurement resolution needs.

## 4. Conclusions

To analyze the effect of all possible micro-force perturbations and temperature fluctuation on an interferometer for the future of the Taiji program, we proposed a simulation modeling method and experimentally verified it, trying to establish the relationship between the noise source and the interference result. The Taiji-1 interferometer model is the concern of this paper, which is in contact with the satellite via six specific contact surfaces. Hence, taking the perturbation analysis of one of six surfaces as an example, the transfer function can be established through modeling simplification, thermal or mechanical disturbance simulation, optical simulation, and post-processing analysis. For micro-force perturbation noise analysis, consistent results are obtained between the linear coefficients of the numerical and experimental results with an error of 0.53%. It provides an efficient and simple way to eliminate the micro-force perturbations for as long as the acquisition of the force distribution of all contact surfaces and the corresponding transfer functions. For temperature fluctuation noise analysis, the nonlinear relationship obtained from simulation process has been verified experimentally with a good consistency. With the help of the transfer function, the interferometric result shows a significant noise suppression effect. Altogether, the simulation modeling method proposed in this paper is effective and expected to be useful for the suppression of noise sources, including force perturbations and temperature fluctuation, and furthers improvement in the measurement accuracy for the future of the Taiji program.

## Data Availability

The data that support the findings of this study are available from the corresponding author upon reasonable request.
